# Gas Chromatography—Fourier Transform Infrared Spectroscopy for Unambiguous Determination of Illicit Drugs: A Proof of Concept

**DOI:** 10.3389/fchem.2020.00624

**Published:** 2020-07-24

**Authors:** Tania M. G. Salerno, Paola Donato, Giampietro Frison, Luca Zamengo, Luigi Mondello

**Affiliations:** ^1^BeSep S.r.l., c/o Department of Chemical, Biological, Pharmaceutical and Environmental Sciences, University of Messina, Messina, Italy; ^2^Department of Biomedical, Dental, Morphological and Functional Imaging Sciences, University of Messina, Messina, Italy; ^3^Laboratory of Environmental Hygiene and Forensic Toxicology, DMPO Department, AULSS 3, Venice, Italy; ^4^Department of Chemical, Biological, Pharmaceutical and Environmental Sciences, University of Messina, Messina, Italy; ^5^Chromaleont S.r.l., c/o Department of Chemical, Biological, Pharmaceutical and Environmental Sciences, University of Messina, Messina, Italy; ^6^Research Unit of Food Science and Nutrition, Department of Science and Technology for Humans and the Environment, Campus Bio-Medico University of Rome, Rome, Italy

**Keywords:** gas chromatography, Fourier Transform Infrared Spectroscopy, solid deposition interface, illicit drugs, synthetic cannabinoids, forensic analysis

## Abstract

The increasing number of synthetic molecules constantly introduced into the illicit drug market poses a great demand in terms of separation and identification power of the analytical tools. Therefore, forensic laboratories are challenged to develop multiple analytical techniques, allowing for the reliable analysis of illicit drugs. This goal is accomplished by means of spectroscopy measurements, usually after a separation step, consisting of liquid (LC) or gas (GC) chromatography. Within the wide range of hyphenated techniques, the coupling of GC to Fourier Transform Infrared Spectroscopy (FTIR) provides a powerful identification tool, also allowing discriminating between isobars and isomers. In this research, the effectiveness of GC-FTIR is demonstrated, in achieving structure elucidation of 1-pentyl-3-(1-naphthoyl)indole, commonly known as JWH-018, a synthetic cannabinoid identified as component of illegal “incense blends.” Moreover, solid deposition FTIR enabled for boosting the sensitivity of the technique, over conventional flow (light pipe) cells, scaling down the limit of identification to the ng scale. Calibration curves for JWH-018 standard were obtained in the 20–1,000 ng range, and the limit of detection and limit of quantification were assessed as equal to 4.3 and 14.3 ng, respectively. Finally, the proposed methodology has been adopted for the identification of active principles in a real “street” sample seized by the law enforcement, consisting of an herbal matrix containing four different synthetic cannabinoids belonging to the JWH class. The correct identification of such compounds, with a high degree of chemical similarity, demonstrated the usefulness of the proposed approach for reliable analysis of complex mixtures of illicit drugs, as viable alternative to widespread mass spectrometry-based approaches.

## Introduction

Since their appearance in the illicit drug market, the number of new psychoactive substances (NPS) is growing at an alarming fast rate; as a consequence, the task of analysis and identification of NPS is posing a big challenge for forensic scientists on one side, and regulatory bodies, for the design and delivery of effective evidence-based responses to drug problems (Zuba, 2014; Lee et al., 2019).

In its latest report, the European Monitoring Center for Drugs and Drug Addiction (EMCDDA) revealed a market that is both resilient and reflective of developments taking place at the global level; the value of the NPS market is unknown actually, however 55 new substances were reported to the European Union Early Warning System (EWS) in 2018, bringing the total number of NPS monitored to 731 (EMCDDA, 2019). Undoubtedly, the shaping and implementation of policy responses and law enforcement activity have contributed to slow-down in appearance of NPS, with respect to the previous decade. However, NPS continue to pose serious cross-border threats to health, with potent synthetic opioids (mainly fentanyls), synthetic cannabinoids and designer benzodiazepines appearing on the market, associated with reports of health emergencies and deaths. Moreover, drug overdoses are commonly associated with the intake (deliberate or not) of multiple substances and thus, health threats and diagnosis may be overlooked without the disposal of adequate forensic and toxicological data. As a consequence, introducing comprehensive screening and increasing the reliability of testing is a central focus for many countries, who have made significant investments in this area. Unfortunately, drug designers are working incessantly to synthesize non-controlled analogs of the drugs of abuse, aiming to get around the existing anti-drug laws, by introducing slight modifications to the chemical structures (UNODC, 2018; Kraenenburg et al., 2019). The constant introduction of new drugs in turn creates a need for reference material to confirm structural elucidation of uncommon or newly encountered substances (Brandt et al., 2014).

In this context, researchers have put considerable efforts in developing advanced chromatographic instrumentation and techniques, aiming to achieve reliable identification of organic compounds in complex mixtures. Approaches based on high-resolution gas chromatography coupled to mass spectrometry (HRGC-MS) are the workhorse analytical tool employed in forensics laboratories, affording the selectivity and sensitivity required for most analytes commonly encountered in seized drugs; however, these hyphenated techniques presents inherent weak points which relate to both the GC and MS counterparts (International, 2015, 2017; Scientific Working Group for the Analysis of Seized Drugs (SWGDRUG), 2019). The data afforded by GC-MS are in fact affected by measurement uncertainties, of different magnitude and sources, of which analysts must be aware: first the uncertainty of measurement for GC retention time, expressed as absolute or relative time units (compared to a known reference standard). Second, the uncertainty of measurement of relative abundances of MS ions obtained by electron impact (EI) ionization; in both cases, specific acceptance criteria recommended by different governing bodies are not uniform (Davidson et al., 2018; Kelly and Bell, 2018).

Whereas, generally accepted as the gold standard of forensic drug analysis, yet GC-MS in some cases suffers from clear limitations, for the identification of co-eluting regioisomeric forms of synthetic drugs of identical elemental composition and yielding identical fragmentation patterns. In some circumstances, positional isomers, and diastereomers may be separated chromatographically, but identification cannot be attained, univocally, on the sole basis of the retention behavior. Whenever structural assignment is mandatory, a further analytical step may be required, consisting of compound isolation or targeted organic synthesis (followed by purification/concentration, eventually) prior to further characterization (Abiedalla et al., 2019; Kraenenburg et al., 2019).

To this concern, the combination of high-resolution gas chromatography (HRGC) to Fourier Transform Infrared Spectroscopy (FTIR) provides a unique tool, through the combination of high efficient separation, and highly specific identification. GC-FTIR allows to quick identifying functional groups in unknown substances, based on the retention behavior of the analytes and the IR absorption bands. Relying on distinct chemical properties, IR may well complement the information afforded by mass spectrometry (MS), in achieving structural identification of volatile and semi-volatile molecules. Moreover, by measuring small energy differences based on rotational and vibrational amplitudes between individual molecular bonds, FTIR spectroscopy enables to overcome one limitation of MS detection, in discriminating regioisomeric compounds (Kempfert, 1988; Almalki et al., 2019).

First attempts to interface a gas chromatograph to IR spectroscopy date back to four decades ago (Griffiths et al., 2008); however the real milestone came in the late 1960s, with the replacement of conventional gratings or dispersive elements with interferometers and FT mathematics (Low and Freeman, 1967). Though exploiting the clear advantage of speed of analysis, yet those hyphenated instruments used high-capacity GC columns and were operated in the stopped-flow mode (Low, 1971; Shaps and Varano, 1977). A flow-through gas cell, known as the light pipe (LP), was used as an interface to deliver vapor-phase IR spectra of eluting solutes (Visser, 2002), with the addition of a flow of make-up gas to compensate for the greater internal diameter of the LP device over that of the GC column (typically, 1.5 vs. 0.32 mm i.d.). In such a way, the chromatographic resolution was kept, even at the cost of longer residence time of the analytes in the interface and, thus, a decrease in the sensitivity. The latter was further impaired by the higher temperature required in the interface for the less volatile GC components, creating background noise in the spectrum (Brown et al., 1985). Indeed, the coupling of GC and FTIR has always posed the need to compromise between sensitivity, and speed. The narrow bandwidths of GC peaks often did not allow for adequate detector sampling, to record a useful spectrum; on the other hand, the amount of analyte required in most cases overwhelmed the GC column capacity. Later on, sample trapping techniques have been developed, aiming to achieve lower detection limits than those afforded by LPs: the matrix isolation (MI) interface (Reedy et al., 1985), and the direct deposition interface (DD) (Fuoco et al., 1986). Both consisted of cryogenic devices allowing for mobile-phase elimination by trapping the GC-separated compounds eluting from the sub-ambient temperature to 11 K (MI) or 77 K (DD). All cold trapping interfaces rely on the use of high vacuum (needed to prevent interferences from environmental water and carbon dioxide), and leak-tight interface housing. In MI, also a cage of 1–2% of argon is added to the carrier gas for freezing the analytes. A critical comparison between light-pipe and sample trapping interfacing methods has been made by Schneider and Demirgian (1986), also discussing *pros* and *cons* in terms of applicability to sample analysis. The light-pipe interface provided a relatively quick and inexpensive way to obtain library-searchable vapor-phase spectra, in real time and in a non-destructive way, from GC eluted components. Whereas, MI interfaces implied a two-step process, since collection of IR spectra from the trapped analytes occurred after the collection was completed. In DD interface, the immobilized spots pass through an external IR beam a few seconds after the deposition, and multiple scans can be taken, as long as the cryogenic conditions are maintained. Both deposition techniques afford at least two orders of magnitude more sensitivity than the LP devices, with the resolution increased from 8 to 4 cm^−1^. As a result of the sharper absorption bands compared with those obtained from free rotating molecules in the gas-phase, also the specificity was increased, in terms of differentiation between closely related molecules. Such features pave the way for the use of solid deposition GC-FTIR as a viable alternative to GC-MS approaches in forensic laboratories, for the reliable identification of NPS such as synthetic cathinones, cannabinoids, phencyclidine analogs, etc.

Originally developed for achieving the desired selectivity toward the cannabinoid receptors CB1 and CB2, synthetic cannabinoids or cannabimimetics were used in the clinical practice to deliver high therapeutic activity (anti-inflammatory and analgesic properties) from unwanted side effects (psychotropic activity). Unfortunately, the information generated by the scientific community has been promptly misused by clandestine laboratories, and these compounds have quickly found their way into the recreational drug market. The detailed pharmacological activities of these analogs are not known, which makes easy access and use of these drugs very dangerous to human health; moreover, synthetic cannabinoids are typically full agonists on the CB1 receptor, thus leading to maximum activation, even at significantly lower doses. Besides their higher potency with respect to the conventional drugs, long half-lives, and formation of active metabolites represent additional hazards deriving from illegal use of cannabimimetics (Bretteville-Jensen et al., 2013; ElSohly et al., 2014). The latter encompasses a wide range of chemical structures, and new analogs are constantly introduced on the market after the preceding drug comes under regulation; this poses additional challenges to drug law enforcement and forensic scientists. Analytical techniques developed so far for the detection and/or quantification of synthetic cannabinoids include colorimetric, immunochemical, and chromatographic methods (Namera et al., 2015).

In this research, the feasibility of using GC-FTIR with solid deposition of the analyte is shown, to achieve unambiguous structure elucidation of 1-pentyl-3-(1-naphthoyl)indole, commonly known as JWH-018, a synthetic cannabinoid identified as component of illegal “incense blends.” Furthermore, the results obtained from the analysis of a seized sample containing several synthetic cannabinoids were compared to those afforded by GC-EI-MS, in terms of identification of unknown components. Detection and identification of unknown NPS in real samples is a major concern when legal or regulatory issues are involved; quantification of targeted analytes may be required, eventually. In a similar way as for MS-based identification, IR spectral data are searched into commercial or custom-made libraries, containing hundreds to thousands IR spectra of standard compounds. When reference materials are measured one at the time, in pure form or constant matrix, then LOD would be sufficient to describe the performance of the measuring system. In this study, another validation parameter was investigated, i.e., the limit of identification (LOI), defined as the lowest analyte concentration that yields a library searchable IR spectrum. In this concern, LOI is related to LOD in that a detectable signal is entailed, but this must also allow for a correct identification to be made, from a defined database. Most often overlooked in similar studies, LOI is a key parameter in determining the possibility for reliable identification of an unknown compound, contained at a certain amount in a given sample, and often in the presence of a noisy background. In a very straightforward way, LOI is sometimes estimated by simply doubling the LOD value (Lanzarotta et al., 2017).

## Materials and Methods

### Standards and Chemicals

Ethyl acetate and methanol pure grade GC-MS solvents were obtained from Merck Life Science (Merck KGaA, Darmstadt, Germany). The following certified reference material (purity ≥98%): 1-naphthalenyl(1-pentyl-1H-indol-3-yl) -methanone (JWH-018), 1-naphthalenyl(1-hexyl- 1H-indol-3-yl)-methanone(JWH-019), 1-naphthalenyl(1-butyl-1H-indol-3-yl) -methanone(JWH-073), were purchased from Cayman Chemical (Ann Arbor, MI, USA) as 1 mg/mL solutions.

### Standard and Sample Preparation

Working solutions of JWH-018 in ethyl acetate were prepared in the concentration range 10–1,000 μg/mL (six concentration levels: 10, 20, 50, 100, 500, 1,000) and analyzed in triplicate. For construction of the calibration curve and for determining the experimental limit of identification (LOI) and the limit of quantification (LOQ), linearity was found in the 20–1,000 μg/mL range. A vegetable matrix sold illegally (and seized in northern Italy) suspected to contain synthetic cannabinoids was extracted at ambient temperature through sonication with methanol (47 mg sample in 1 mL of solvent) and filtered prior to injection into the GC-FTIR system.

### Gas Chromatography

GC analyses were performed on a Nexis GC-2030 gas chromatograph equipped with AOC-20i auto sampler (Shimazdu, Kyoto, Japan). All GC separations were carried out on a Supelco SLB-5ms column (30 m L × 0.25 mm i.d., 0.25 μm d_f_) (Merck KGaA, Darmstadt, Germany), under the same conditions. Injections were performed in splitless mode (1.50 min sampling time), with an injection volume of 1 μL and an injector temperature of 280°C. Helium (purity 99.99%) was used as carrier gas in constant linear velocity of 30 cm/s, and a pressure of 109 kPa at the begin of the ramp temperature. The oven temperature was programmed as follows: 100°C for 2 min, then ramp to 350°C at 15°C/min. The final temperature was held for 5.0 min, resulting in total GC run times of 24.0 min. The end of the column was connected to a deactivated fused silica capillary through a micro Siltite μ-union (Trajan Scientific and Medical, Milton Keynes, UK) and inserted into a heated transfer line connected to the IR interface. The transfer line and restrictor temperatures were set at 280°C. Blanks were run in between samples to assure that the liner and column were free of contamination.

### GC-FTIR Interface

The transfer line exiting the GC oven was connected into a DiscovIR solid phase FTIR detector (Spectra-Analysis Instrument Inc., Marlborough, MA, USA). The restrictor was positioned directly above a ZnSe disk, which was chilled down to −50°C by means of liquid nitrogen, and cleaned daily with acetone. The DiscovIR FTIR instrument was equipped with a Mercury-Cadmium-Telluride (MCT) cryogenically cooled detector. Solid phase IR spectra of the GC eluted compounds were recorded in real time from 100 μm × 100 μm spots in the 650–4,000 cm^−1^ range, with a resolution of 4 cm^−1^ and a scan rate of 2 Hz, at a disc rotation speed of 3 mm/min.

### GC-FTIR Data Acquisition and Processing

The GC instrument was controlled by the LabSolution software (Shimazdu, Kyoto, Japan). GC-FTIR data acquisition and processing were performed using the Thermo Galactic GRAMS/AI spectroscopy and chromatography software ver. 9.3 provided within the instrument. Compounds were identified through the library search program (Spectral ID), using a first derivative correlation algorithm, by comparison within a custom-made solid phase IR spectral library containing IR spectral data of around 600 synthetic cannabinoids and other NPS (namely, Controlled and Prohibited Substances ver. 1.0). The results expressed by the software in 0–1 units (were 0 represents the maximum value for similarity or quality score), was converted for simplicity and uniformity with other search software into 1–100% units (where 100 represents the maximum quality score), by using the formula: Quality score = (1-GRAMS value)^*^100.

### GC-MS

GC-MS analyses were performed on a GCMS-QP2020 NX instrument, equipped with AOC-20 auto sampler and EI source (Shimazdu, Kyoto, Japan). The experimental conditions were the same as those employed for GC-FTIR analyses, except for the injection, which was performed in split mode (1:50), with an injection volume of 0.3 μL (injector temperature of 280°C). MS parameters were as follows: full scan mode in the 40–650 m/z range, ion source temperature: 250°C, interface temperature: 200°C.

Blanks were run in between samples to assure that the liner and column were free of contamination.

Data acquisition and processing was performed by LabSolutions GCMSsolution software ver. 4.41 (Shimazdu, Kyoto, Japan). Compounds were identified by comparison within SWGDRUG MS Library ver. 3.6 (available at http://www.swgdrug.org/ms.htm), containing over 3,000 EI mass spectra of drugs and drug-related compounds.

## Results and Discussion

The use of GC-FTIR as an effective tool for forensic drug identification has been already demonstrated 30 years ago, in terms of specificity needed to differentiate between closely related isomers, including cocaine/pseudococaine, phentermine/metamphetamine (Kempfert, 1988). In contrast to data afforded by widespread MS detection, the uniqueness of IR spectra allows to quickly discriminate between isomers other than optical isomers without the need for preliminary purification/derivatization, as proven for a number of different drug categories, including cannabinoids (Smith et al., 2014, 2018; Belal et al., 2018; DeRuiter et al., 2018; Lee et al., 2019). Since the disclosure of their existence in herbal mixtures (Auwarter et al., 2009), N-alkyl indole-3-carbonyl derivatives targeting cannabinoid receptors have been largely abused, and have accounted for a major portion of new psychoactive substances put illegally on the market. Many of these synthetic molecules result from modifications of the parent molecule1-naphthalenyl(1-pentyl-1H-indol-3-yl)-methanone shown in Table 1, commonly known as JWH-018, such as: the substitution of the indole core ring with other systems (pyrrole, indazole and carbazole), the introduction of acyl groups different than naphthalenyl at the 3-position of indole ring, or the modification of the alkyl chain at the 1-position (different chain length, chain branching, fluoroalkyl groups). The obvious goal of these slight structural changes is to create analogs which are regarded as beyond the scope of the regulation.

**Table 1:** Akyl-3-acyl-indole derivatives of the JWH series.

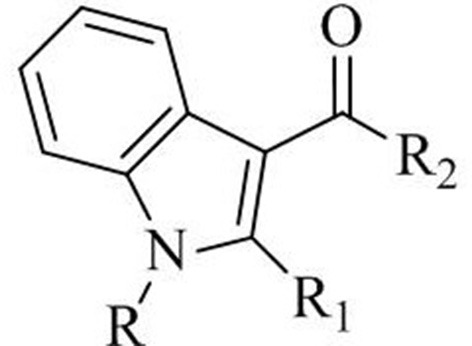				
**Common name**	**Formula**	**R**	**R_**1**_**	**R_**2**_**
JWH-018	C_24_H_23_NO	*n-Pentyl*	*H*	*1-Naphtyl*
JWH-019	C_25_H_25_NO	*n-Hexyl*	*H*	*1-Naphtyl*
JWH-073	C_23_H_21_NO	*n-Butyl*	*H*	*1-Naphtyl*
JWH-020	C_26_H_27_NO	*n-Heptyl*	*H*	*1-Naphtyl*
JWH-015	C_23_H_21_NO	*n-Propyl*	*CH_3_*	*1-Naphtyl*
JWH-250	C_23_H_21_NO	*n-Pentyl*	*H*	*2-Methoxy-benzyl*
JWH-019 N (6-fluorohexyl) isomer	C_25_H_24_FNO	*5-Fluoro-n-pentyl*	*H*	*2-Iodo-phenyl*
JWH-018 N (3-methylbutyl) isomer	C_24_H_23_NO	*n-3-Methylbutyl*	*H*	*1-Naphtyl*

### Optimization of Solid Phase GC-FTIR

**Figure 1:**
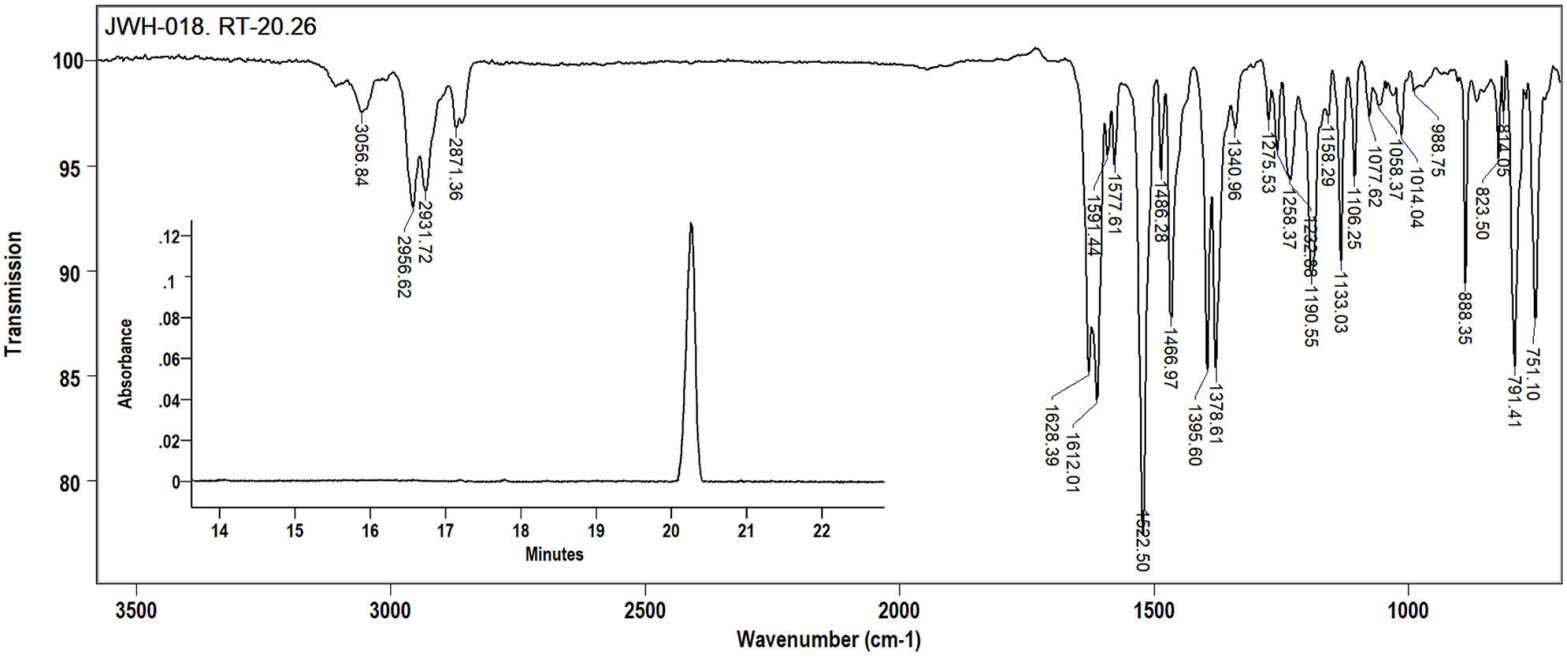
Solid-phase mid-IR spectrum for JWH-018 (1-naphthalenyl(1-pentyl-1H-indol-3-yl)-methanone) at 4 cm^−1^ resolution. Inset: the Grams-Schimdt reconstructed GC-FTIR chromatogram (SLB-5ms, 30 m L × 0.25 mm i.d., 0.25 μm d_f_).

The first step of this research consisted in optimization of the analysis conditions for JWH-018, with regard to the parameters affecting the hyphenation of the two techniques, and FTIR detection as the end goal. The calculation of IR response vs. time was done with the use of the standard software package which utilizes a Gram-Schmidt reconstruction (GSR), resulting in the GC-FTIR chromatogram in Figure 1 (inset). It can be appreciated how the result of this reconstruction process closely resembles that of a total ion chromatogram from a GC-MS system.

**Figure 2:**
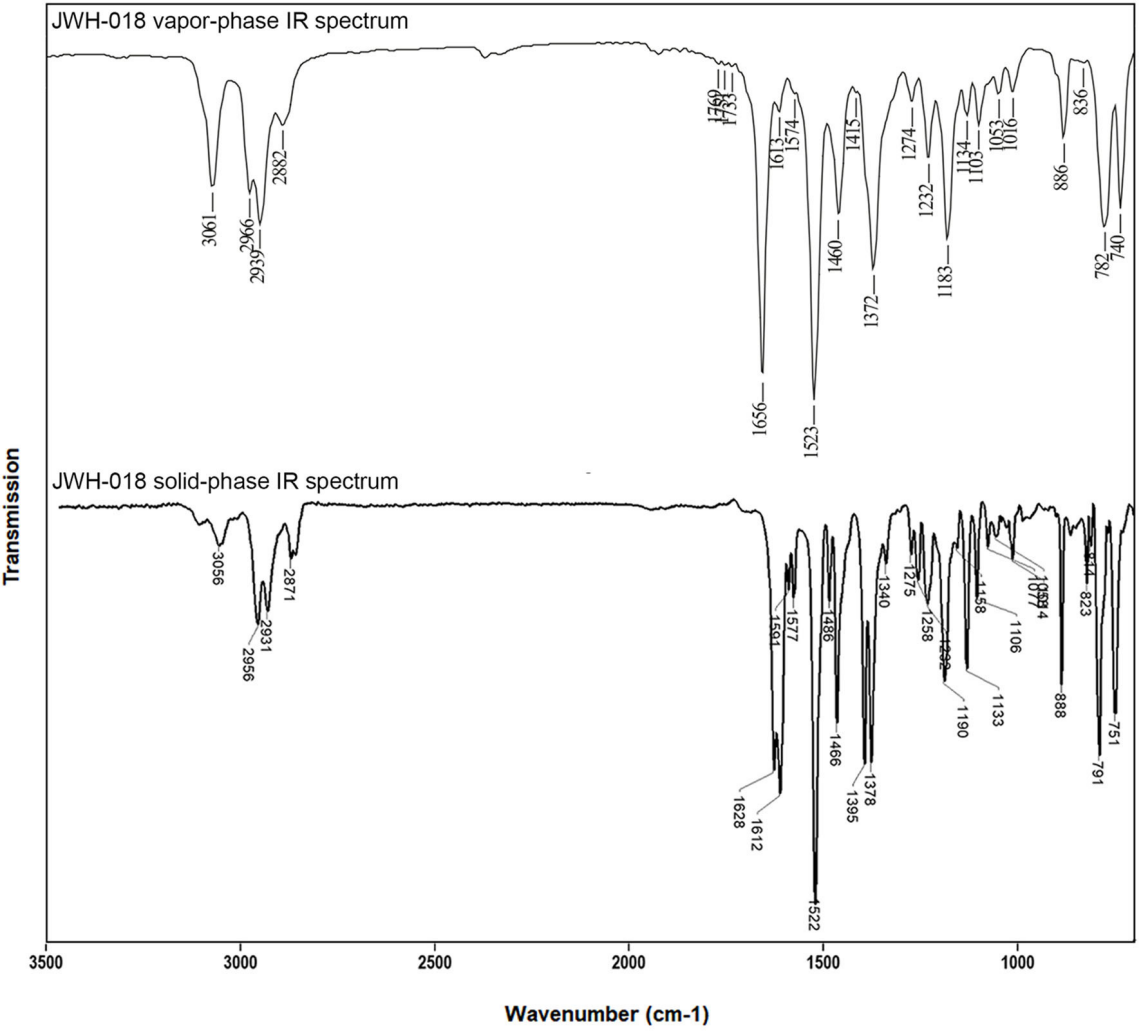
Vapor-phase **(top)** and solid-phase **(bottom)** mid-IR spectra for JWH-018 (1-naphthalenyl(1-pentyl-1H-indol-3-yl)-methanone), at a resolution of 8 and 4 cm^−1^, respectively. Top part of this figure has been reprinted from Spectrochimica Acta Part A: Molecular and Biomolecular Spectroscopy, 196, Lewis W. Smith, Amber Thaxton-Weissenfluh,Younis Abiedalla, Jack De Ruiter, Forrest Smith, C. Randall Clark, Correlation of vapor phase infrared spectra and regioisomeric structure in synthetic cannabinoids, page 10, Copyright 2018, with permission from Elsevier.

The overall performance of the GC-FTIR technique relies on the deposition of the GC-eluted compounds contained in a carrier gas stream onto the ZnSe disc; thus, one main factor affecting the quality of an IR spectrum is the disc temperature, which can be adjusted by the amount of liquid nitrogen supplied from a Dewar. If insufficient chilling is provided to the disc, trapping of the analytes would be incomplete, with a detrimental effect on the sensitivity of the technique, to a different extent depending on the volatility of the molecules. For the low volatile compounds under study, the maximum analyte recovery in the solid state was obtained at a disc temperature of −50°C. Under the selected experimental conditions, JWH-018 was eluted from the 30 m SLB-5ms column at a retention time just above 20 min as shown in the inset in Figure 2. One microliter splitless injection of the ethyl acetate JWH-018 standard solution, corresponding to 1 μg injected on column, gave a chromatographic base peak width of 26 s (52 data points), at an optimum disc rotation speed of 3 mm/min. A proper visual description of the analyte peak requires a discrete number of data points across the same peak, as dictated by the detector acquisition rate and the chromatographic peak width; in temperature-programmed GC analyses, the latter may be affected by large carrier-gas velocity changes, to a variable extent. To this concern, matching the disc rotation speed to the amount of analyte being delivered by the deposition tip is crucial to proper interrogate the deposited solid, avoiding losses in the sensitivity if the time required for complete analyte deposition is not allotted.

On the other hand, slowing down the disc rotation rate excessively would inevitable come at the cost of sacrificed (chromatographic) resolution, which is of utmost concern in FTIR spectra.

The FTIR transmittance spectrum of JWH-018, recorded in the 650–4,000 cm^−1^ at a resolution of 4 cm^−1^ is shown in Figure 2. In the 3,000 wavenumber region, two medium intensity IR bands at 2,955 and 2,931 cm^−1^ and a weaker band at 2,872 cm^−1^ arise from the aliphatic C-H stretching, while a single minor band at 3,059 cm^−1^ represents the aromatic C-H stretching. A strong carbonyl absorption band is visible in the wavenumber region around 1,620 cm^−1^; noticeably this C-O stretching appears as a doublet, due to the higher spectral resolution of the GT-FTIR (solid phase) interface employed, compared to that of light-pipe (gas phase) devices employed elsewhere (Smith et al., 2018). As absorption bands are broadened by molecular rotation or intermolecular forces, the fine structure of the IR spectrum is lost; moreover, the high temperatures required in the light-pipe method cause the absorption bands to be broadened and the spectral specificity reduced. The differences in IR data quality afforded by the two different techniques can be easily appreciated in Figure 2, showing a visual comparison of mid-IR spectra for JWH-018 by using LP and solid phase GC-FTIR interfaces.

Calibration curves for JWH-018 were obtained from triplicate injections of five concentration levels in the 20–1,000 mg/mL range, by plotting the chromatographic peak area as a function of the analyte concentration. The IR response vs. amount injected was linear over the range investigated (linear regression: y = 4E-05x - 0.0004, *R*^2^ = 0.996) and the figures of merit determined in terms of limit of detection (LOD) and limit of quantification (LOQ), with an average e relative standard deviation (RSD%) per calibration point of 5.42. A LOD value of 4.3 ng was defined, as the sample amount yielding a peak equal to three times the peak-to-peak noise level without any post-run treatment, thus mimicking the situation in which a complete unknown is to be identified, in a real sample application. The low end of the linear detection range (LDR) was defined as the LOQ value of 14.3 ng, as the sample amount yielding a peak equal to 10 times the peak-to-peak noise level. The high end of the LDR could be determined as the highest concentration retaining linearity, without overloading the inlet liner or column, resulting in poor peak shape or carryover between consecutive runs. However, this figure could be not determined experimentally, since it would be higher than the concentration of the standard solution provided by the manufacturer.

It must be specified at this point, that the LOD and LOQ values for the solid-phase GC-FTIR technique were estimated from plots of the chromatographic peak area vs. analyte concentration, in a non- targeted way. This approach more fits the need for unknown drug identification and quantification in real samples; however a targeted approach based on absorbance measurement of the most intense IR peak as a function of time would yield different figures of merit, as regards increased selectivity and sensitivity (Lanzarotta et al., 2017).

### Identification Through Solid-Phase Library Search

The sharp absorption bands in FTIR spectra obtained from solid deposited analytes give the ability to differentiate between similar compounds, and may well complement the type of data provided by GC-MS analysis, in achieving reliable identification. However, results based on qualitative review of the IR data by the analyst are heavily subjective and may lead to different outcomes; for this reason, it is desirable to implement more objective algorithm-based criteria, based on quantitative data evaluation. Hereby, the quality score (QS) or quality match factor (QMF) was adopted, as an unbiased criterion to differentiate between IR spectra and achieve unambiguous compound identification upon library search.

**Figure 3:**
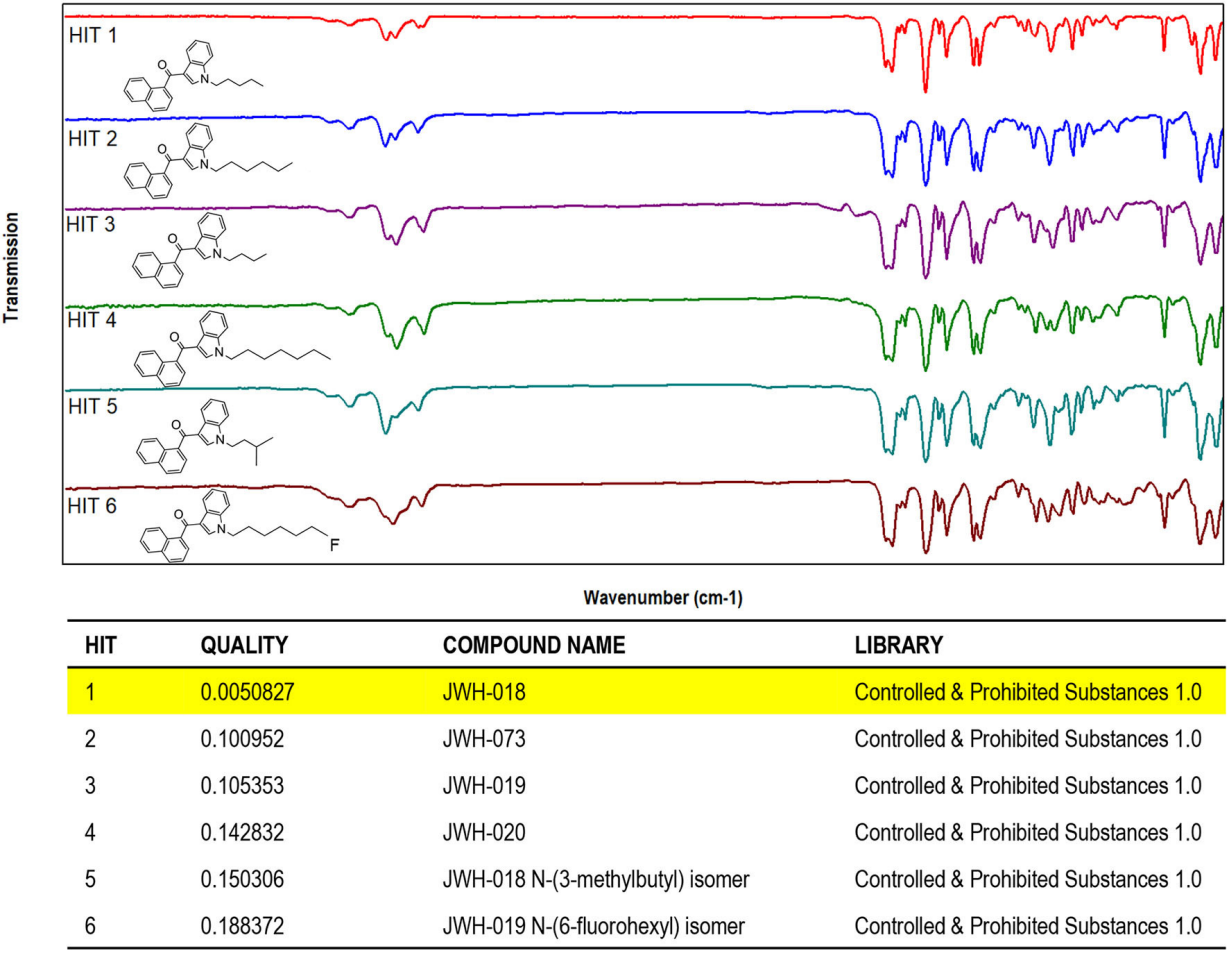
Software window showing the library search results obtained for JWH-018 IR spectral data. **(Top)** IR spectra of JWH and its structural analogs (Hits 1–6). **(Bottom)** QMF obtained for JWH-018 against correct and incorrect matches. Quality is expressed in 0–1 units (with 0 representing the maximum value for similarity or quality score).

The results obtained by searching the IR spectral data for JWH-018 into a custom-made library containing IR spectral data of synthetic cannabinoids and other psychoactive substances (supplied by the vendor) demonstrated that the compounds can be differentiated and identified with no ambiguity, through the evaluation of QMF (Figure 3).

**Figure 4:**
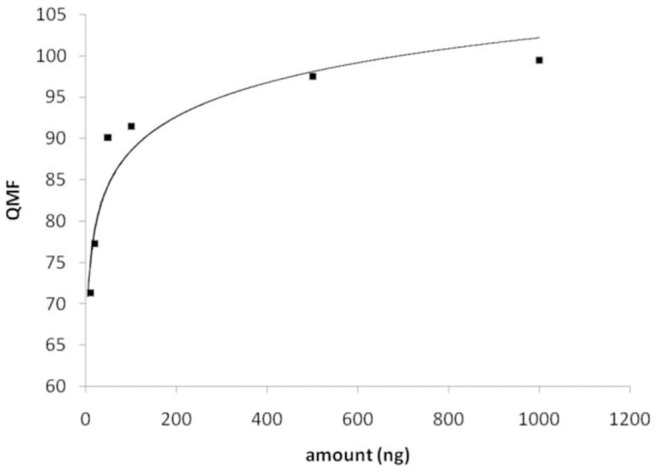
Plot of the QMF values obtained for library search of JWH-018, as a function of the amount measured.

As it can be seen in the results from library search in Figure 3 (bottom), JWH-018 was correctly identified with a similarity around 0.005, which corresponds to a QMF of 99.5% [QMF= (1-GRAMS value)^*^100]. Noticeably, JWH-018 gave a QMF below 90% (ranging from 89.9 to 81.2%) when it was matched to the wrong compounds, including one regioisomer (Hit 5). A visual comparison of the IR spectra of Hits 1–6, shown in the bottom part of Figure 4, reveals a high degree of similarity, the only differences being related to the different length of the N-alkyl chain. These are reflected in the aliphatic C-H stretching bands in the 2,900 cm^−1^ region and C-H bending bands in the wavenumber region below 1,200 cm^−1^. The terminal halogen substituted derivative (6-fluorohexyl, hit 6) shows changes in the aliphatic triplet pattern around 3,000 cm^−1^ to almost a single band with weak shoulder inflections, due to the loss of the terminal methyl group of the N-alkyl tail substituent; moreover C-F stretching bands are in the fingerprint region of 1,000–1,300 cm^−1^. Nonetheless, these small spectral differences are successfully captured by the QMF values obtained for the different matches, demonstrating the usefulness of the technique to differentiate between closely related molecules of the JWH series. Among all the JWH-018 analogs, the JWH-073 and JWH-019 gave the closest match (Hits 7 and following gave QMF below 80% and are not shown), thus they were analyzed separately to further validate the results. The resulting QMF of the three compounds matched again each other are presented in Table 2; thus, a QS or QMF of 0.1 (90%) could be assumed for a correct identification, given the high specificity of the spectral data, to reduce the likelihood for false positives and increase confidence in the results.

**Table 2:** QMF for JWH-018 and its related compounds.

**Compound**	**QMF to the analog compound**		
	**JWH-018**	**JWH-073**	**JWH-019**
JWH-018	**99.5**	89.4	86.2
JWH-073	89.9	**96.9**	83.8
JWH-019	89.5	81.8	**94.8**

Results rendered by algorithm-based search criteria in 0–1 units were converted into 1–100% units [QMF = (1-GRAMS value)*100]. In bold are the QMF of the compound itself.

Hereby, as a more trustworthy approach, the IR spectra recorded for JWH-018 solutions at concentration levels investigated for the LDR, were searched into the solid-phase FTIR library. A plot of the QMF values obtained of the compound itself, showed a logarithmic dependence from the amount measured, as in Figure 4. Based on the criterion established before, the minimum amount of substance yielding a library searchable IR spectrum (QMF ≥ 90%), is just below 50 ng.

### Solid Phase GC-FTIR and GC-EI-MS Analysis of a Seized Sample

NPS are increasingly sold and consumed as mixtures of several different active principles, whose pharmacodynamics and adverse effects are almost unknown, and with large intra- and inter-product concentration variabilities. This poses a challenge for forensic laboratories, as they are frequently asked to promptly identify unknown substances, both for the court and/or to orient emergency treatments in intoxication cases (Zamengo et al., 2014).

To assess the applicability of the technique for analysis of a real sample, a vegetable matrix, suspected to contain one or more synthetic cannabinoids, was extracted by sonication and injected into the GC-FTIR and GC-EI-MS systems; analyses were made in triplicate.

**Figure 5:**
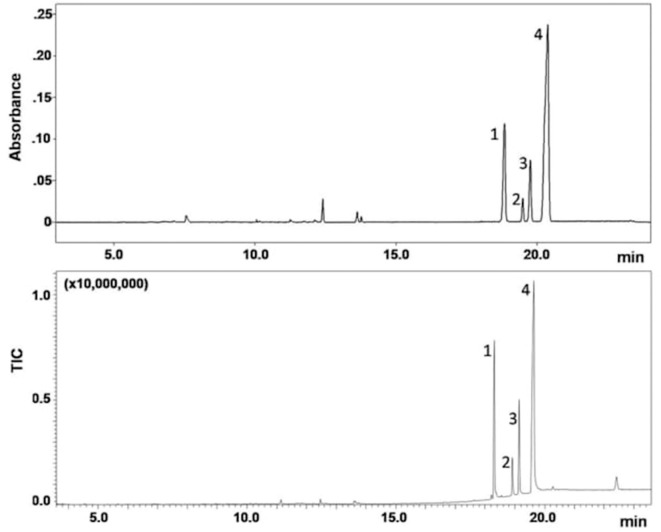
GC-FTIR **(top)** and GC-EI-MS **(bottom)** analysis of a seized sample. Column: SLB-5ms (30 m L × 0.25 mm i.d., 0.25 μm d_f_), carrier gas: helium at 1.1 mL/min (30 cm/s, 109 kPa); oven: 100°C for 2 min, to 350°C at 15°C/min (held for 5.0 min); injection: 1 μL splitless (GC-FTIR) or 0.3 μL split 1:50 (GC-MS) at 280°C. Peak identification: JWH-250 (1), JWH-015 (2), JWH-073 (3), JWH-018 (4).

The GC-FTIR and GC-MS chromatograms of the extracted drug sample are shown in Figure 5. Four major components belonging to the JWH series were separated on a 30 m length of non-polar bonded and highly crosslinked silphenylene polymer column, virtually equivalent in polarity to a poly (5% diphenyl/95% dimethyl siloxane) column, which is commonly applied in forensic drug analysis. These synthetic cannabinoids were later identified as: 1-(1-pentyl-1H-indol-3-yl)-2-(2-methoxyphenyl)-ethanone (JWH-250), (2-methyl-1-propyl-1H-indol-3-yl)-1-naphthalenyl-methanone (JWH-015), (1-butyl-1H-indol-3-yl)-1-naphthalenyl-methanone (JWH-073), and 1-naphthalenyl(1-pentyl-1H-indol-3-yl)-methanone (JWH-018); their chemical structures are given in Table 1.

It is worth mentioning at this point that a method for untargeted analysis was developed, and the GC program temperature optimized to achieve baseline separation of all possible cannabinoid constituents in unknown samples. Moreover, this program allowed for calculation of the Linear Retention Indices of synthetic cannabinoids, upon injection of a C4–C40 alkane reference mixture (data not included in the work). The use of a higher initial temperature, or a much steeper ramp, would allow to speed up elution of the compounds of interest, absolutely; however it was not the scope of this work.

**Figure 6:**
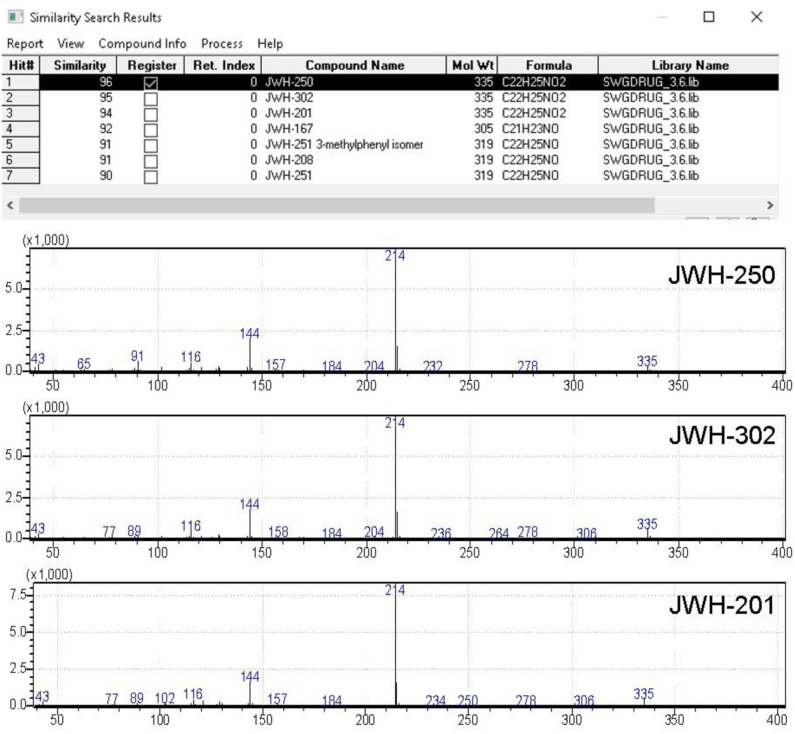
Software window showing the library search results obtained for JWH-250 EI-MS spectral data. QMF obtained for against correct and incorrect matches are shown in column #2.

The GC-MS technique afforded superior resolution and sensitivity within the same analysis time, in part due to faster detector scan rate (100 Hz) with respect to the FTIR counterpart (2 Hz). An additional minor peak showed up in the GC-MS chromatogram after the compounds of interest, at *t*_*R*_ of 22.5 min (Figure 6-bottom), which could not be detected by GC-FTIR. In contrast, the upper GC-FTIR trace reveals three minor peaks, eluting in the 12–14 min retention time window (Figure 5-top), which were not recorded by GC-MS. This latter evidence is, in the authors' opinion, intrinsic to the distinct technologies, in fact FTIR may be regarded as “compound independent,” in that once GC eluted analytes from the tip are deposited as solid spots onto the disk, FTIR spectra may be recorded. In other words, no disparity in ionization may affect the spectral result.

The analysis of street drug samples relies strictly on the specificity of the technique for the target substance of abuse, and thus on the capability to discriminate between a plethora of possible “co-formulants” with nearly identical chemical structures. In this study, the results obtained by GC-FTIR and GC-EI-MS were evaluated, to assess the capabilities of the two techniques, in allowing for ultimate identification of the seized sample components. To this purpose, IR and MS spectral data were searched into a custom-made solid phase FTIR library, containing IR spectral data of around 600 synthetic cannabinoids and other NPS, and the SWGDRUG MS Library ver. 3.6 (available at http://www.swgdrug.org/ms.htm), containing over 3,000 EI mass spectra of drugs and drug-related compounds. The resulting QMF values obtained by the two techniques are listed in Table 3, reported as the correct matches for each compound identified.

**Table 3:** QMF for the compounds identified in a seized sample.

**Compound**	**QMF to the analog compound**	
	**GC-FTIR**	**GC-MS**
JWH-250	98.0	96
JWH-015	91.5	89
JWH-073	95.6	90
JWH-018	96.7	92

Results rendered by algorithm-based search criteria in 0–1 units were converted into 1–100% units [QMF = (1-GRAMS value)*100].

The FTIR spectra obtained by solid-deposition interface in all cases succeeded to discriminate between the different substances, with QMF values ranging from 91 to 98%, thus confirming validity of the criterion of QMF of 90 and above to ascertain unequivocal identification of the correct molecule. The QMF values obtained in a similar way upon library search of MS spectral data were for all the correct matches lower, with a maximum QMF of 96 and a minimum QMF of 89, with the last result being lower than the minimum acceptance criterion.

A further concern in library search is the QMF differential between the correct and the (closest) incorrect matches, in that it allows for confident structure identification to be achieved, even if the spectrum of a target analyte is not included in the specific library. To this regard, the case of the first eluting sample component, labeled as peak #1 at *t*_*R*_ around 18 min (chromatograms in Figure 5), is noteworthy. This compound was identified by library search of EI-MS data, as shown in Figure 6, as JWH-250, with a QMF of 96 (Hit #1). While this would be regarded as quite satisfactory for assessing identification, however it must be noted that Hit #2 and Hit #3, representing the closest incorrect matches for JWH-302 and JWH-201, respectively, shows nearly identical QMF value, *viz*. 95 and 94. In this situation, very little information is available for the specific differentiation of regioisomers having identical nominal and observed masses, and even abundant fragment ions obtained by EI occur at equivalent masses: 1-(1-pentyl-1H-indol-3-yl)-2-(2-methoxyphenyl)-ethanone (JWH-250), 2-(3-methoxyphenyl)-1-(1-pentyl-1H-indol-3-yl)-ethanone (JWH-302), 2-(4-methoxyphenyl)-1-(1-pentyl-1H-indol-3-yl)-ethanone (JWH-201), with Molecular Formula C_22_H_25_NO_2._

**Figure 7:**
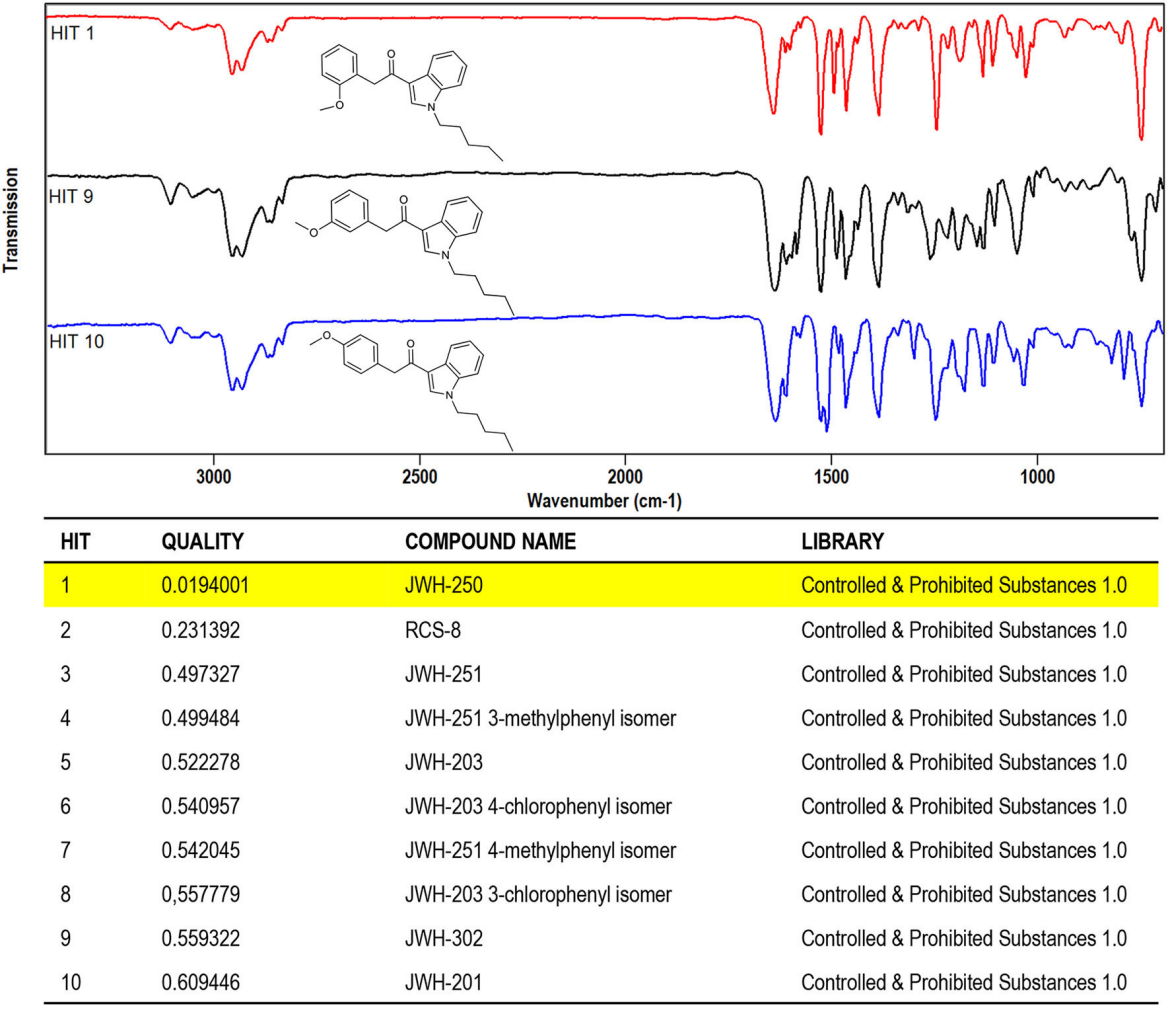
Software window showing the library search results obtained for JWH-250 IR spectral data. **(Top)** IR spectra of JWH-250 and its regioisomers JWH-302, JWH-201 (Hits 9, 10). **(Bottom)** QMF obtained for JWH-250 against correct and incorrect matches. Quality is expressed in 0–1 units (with 0 representing the maximum value for similarity or quality score).

Likewise, the results obtained by library search of solid-phase FTIR data, shown in Figure 7, fully prove the capability of the technique in affording the specificity of information required. It can be seen that regioisomeric compounds ranked as Hit #1(JWH-250), Hit #9 (JWH-302), and Hit #10 (JWH-201), with a QMF value of 98.0 for the correct match, vs. 44.0 and 39.0 for the two incorrect matches (Figure 7-bottom). The high discrimination power of solid-phase FTIR may be appreciated from a visual comparison of the IR spectra stacked in Figure 7 (top), where changes in positional bonding result in unique patterns, especially in the complex fingerprint region, that allow for differentiation among the three regioisomers.

## Conclusions

The effectiveness of solid-phase GC-FTIR is demonstrated, as an alternative tool to widespread MS-based approaches, for achieving unequivocal identification of NPS. The use of a solid deposition interface enabled for boosting the resolution and sensitivity of the technique, over gas phase cells, scaling down the limit of identification to the ng scale. Results obtained from the analysis of an illicit street drug sample showed that GC-MS alone would in this case not afford the reliable identification of the unknown compounds, with the exclusion of the other possible regioisomeric molecules. The lack in MS specificity, along with the likelihood for chromatographic co-elutions, and the possible scarcity of available reference material would make drug identification challenging. Thus, the individual identification of any one of these substances and the exclusion of possible misidentification will depend heavily upon chromatographic methods. Likewise, solid-phase GC-FTIR succeeded in allowing for confident identification of all the compounds in a forensic drug sample, given the ability to eliminate regioisomers as possible interfering or co-eluted substances. This additional specificity of FTIR comes at the price of lower sensitivity (at the ng scale) with respect to the MS counterpart (at the pg scale), nonetheless this may be not a primary concern in forensic science, since illicit drugs usually contain up to milligrams of psychoactive substances.

## Data Availability Statement

The raw data supporting the conclusions of this article will be made available by the authors, without undue reservation.

## Author Contributions

LM: conception and design. TS: acquisition of data. TS, PD, GF, and LZ: analysis and interpretation of data. PD: drafting the article. All authors: contributed to the article and approved the submitted version.

## Conflict of Interest

The authors declare that the research was conducted in the absence of any commercial or financial relationships that could be construed as a potential conflict of interest. The handling Editor declared a past co-authorship with the authors GF and LZ.
